# The Cumulative Cisplatin Dose Affects the Long-Term Survival Outcomes of Patients with Nasopharyngeal Carcinoma Receiving Concurrent Chemoradiotherapy

**DOI:** 10.1038/srep24332

**Published:** 2016-04-13

**Authors:** Hao Peng, Lei Chen, Wen-Fei Li, Rui Guo, Yan-Ping Mao, Yuan Zhang, Fan Zhang, Li-Zhi Liu, Li Tian, Ai-Hua Lin, Ying Sun, Jun Ma

**Affiliations:** 1 Department of Radiation Oncology, Sun Yat-sen University Cancer Center, State Key Laboratory of Oncology in Southern China, Collaborative Innovation Center for Cancer Medicine, People’s Republic of China; 2 Imaging Diagnosis and Interventional Center, Sun Yat-sen University Cancer Center, State Key Laboratory of Oncology in Southern China, Collaborative Innovation Center for Cancer Medicine, People’s Republic of China; 3 Department of Medical Statistics and Epidemiology, School of Public Health, Sun Yat-sen University, People’s Republic of China

## Abstract

The prognostic value of the cumulative cisplatin dose (CCD) remains controversial for patients with nasopharyngeal carcinoma (NPC) receiving only concurrent chemoradiotherapy (CCRT). We retrospectively reviewed 549 consecutive patients with non-metastatic, histologically-proven NPC treated using intensity-modulated radiotherapy (IMRT) at Sun Yat-sen university cancer center. Patient survival between different CCD groups were compared. The cut-off value of pre-treatment plasma EBV DNA (pre-DNA) and CCD based on disease-free survival (DFS) were 1460 copies/ml (AUC, 0.691; sensitivity, 0.717; specificity, 0.635) and 240 mg/m^2^ (AUC, 0.506; sensitivity, 0.526; specificity, 0.538), respectively. Of the entire cohort, 92/549 (16.8%) patients received a CCD ≥ 240 mg/m^2^ and 457 (83.2%) patients, <240 mg/m^2^. For CCD ≥ 240 mg/m^2^ vs. < 240 mg/m^2^, the estimated 4-year DFS, overall survival (OS), locoregional-free survival (LRFFS) and distant metastasis-free survival (DMFS) rates were 89.1% vs. 81.3% (*P* = 0.097), 92.4% vs. 90.0% (*P* = 0.369), 95.6% vs. 91.2% (*P* = 0.156), and 91.3% vs. 88.4% (*P* = 0.375), respectively. For the whole cohort, multivariate analysis identified the CCD was an independent prognostic factor for DFS (HR, 0.515; 95% CI, 0.267–0.995; *P* = 0.048). However, CCD (≥240 mg/m^2^) had no prognostic value in subgroup analysis with stratification by the cut-off value of pre-DNA (P > 0.05 for all rates).

Nasopharyngeal carcinoma (NPC) has an extremely unbalanced age-standardized incidence of 20–50 per 100,000 males in southern China and 0.5 per 100,000 in predominantly white populations of European origin^1^. Due to anatomic constraints and its high radiosensitivity, radiotherapy is the primary and only curative treatment, and the TNM staging system remains the most reliable method for devising clinical treatment strategies and predicting prognosis in NPC^2^. However, the TNM staging system is only based on anatomical features and does not include other effective prognostic factors. Therefore, more prognostic factors should be identified to improve the accuracy of predicting prognosis.

Since the NPC-0099 trial reported chemoradiotherapy was superior to radiotherapy alone^3^, subsequent clinical trials have confirmed the efficacy of chemotherapy in advanced-stage NPC^4^,^5^,^6^,^7^. The National Comprehensive Cancer Network (NCCN) guidelines recommend single-agent 100 mg/m^2^ cisplatin every 3 weeks for three cycles as the main standard treatment for stage II-IVB NPC. In the prospective NPC-0099 and NPC-9901 trials, 100 mg/m^2^ cisplatin was given every 3 weeks to reach a targeted cumulative dose of 300 mg/m^2^^3^,^5^. However, a cumulative dose of 200 mg/m^2^ cisplatin had been reported to be sufficient to yield a beneficial anti-tumor effect in other head and neck cancers^8^ and NPC^9^,^10^. Most recently, Ou *et al.*^11^ revealed a total cisplatin dose of 300 mg/m^2^ was an independent prognostic factor for better distant metastasis and overall survival in local advanced NPC. This controversial results urgently need to be investigated further.

Therefore, we retrospectively assessed the relationship between the cumulative cisplatin dose (CCD) and the long-term outcomes of patients with NPC receiving concurrent chemoradiotherapy (CCRT) and intensity-modulated radiation therapy (IMRT). Moreover, the relationship between the pre-treatment Epstein-Barr virus DNA load (pre-DNA) and cumulative cisplatin dose was explored.

## Methods and Materials

### Patient Selection

We retrospectively reviewed 1811 patients with previously untreated, biopsy-proven NPC with no evidence of distant metastasis treated using IMRT between November 2009 and February 2012 at Sun Yat-sen University Cancer Center. The 549 (30.3%) patients receiving only cisplatin during CCRT were included in this investigation. All experimental protocols were approved by the Research Ethics Committee of Sun Yat-sen University Cancer Center, patient confidentiality was protected at all times, and informed consent was obtained from all patients. All the methods were carried out in accordance with the approved guidelines in this study.

### Clinical Staging

Routine staging included a complete medical history, clinical examination of head and neck, direct fiber-optic nasopharyngoscopy, magnetic resonance imaging (MRI) of skull base and entire neck, chest radiography, whole-body bone scan, abdominal sonography and positron emission tomography (PET)-CT. The tumour-associated markers immunoglobulin A (IgA) antibodies to EBV viral capsid antigen (VCA) and EBV early antigen (EA) and plasma EBV DNA were quantified. All patients had a dental evaluation before radiotherapy and were restaged according to the 7^th^ edition of the International Union against Cancer/American Joint Committee on Cancer (UICC/AJCC) system^12^. All MRI materials and clinical records were reviewed to minimize heterogeneity in restaging. Two radiologists employed at our hospital separately evaluated all scans; disagreements were resolved by consensus.

### Quantitation of Epstein-Barr Virus DNA

Plasma EBV DNA was quantified before treatment using a real-time quantitative PCR assay^13^ that targets the *BamH*I-W region of the EBV genome using the primers 5′-GCCAGAGGTAAGTGGACTTT-3′ and 5′-TACCACCTCCTCTTCTTGCT-3′ and dual fluorescence-labeled oligomer probe 5′-(FAM)CACACCCAGGCACACACTACACAT(TAMRA)-3′. EBV genome sequence data were obtained from the GeneBank sequence database.

## Treatment

### Radiotherapy

All patients received definitive IMRT at our center while immobilized using a custom-made head-to-neck thermoplastic cast and neck support. A high-resolution planning computed tomography scan (Siemens, Plus 4) with contrast was taken from the vertex to 2 cm below the sternoclavicular joint (slice thickness, 3 mm). Target volumes were delineated slice-by-slice on treatment planning CT scans using an individualized delineation protocol that complies with International Commission on Radiation Units and Measurements reports 50 and 62. Prescribed doses were 66–72 Gy at 2.12–2.43 Gy/fraction to planning target volume (PTV) of primary gross tumour volume (GTVnx), 64–70 Gy to PTV of GTV of involved lymph nodes (GTVnd), 60–63 Gy to PTV of high-risk clinical target volume (CTV1), and 54–56 Gy to PTV of low-risk clinical target volume (CTV2). All targets were treated simultaneously using the simultaneous integrated boost technique.

### Chemotherapy

According to institutional guidelines, we recommended radiotherapy alone for stage I disease, CCRT for stage II and CCRT +/− neoadjuvant/adjuvant chemotherapy for stage III-IVA-B. In addition, patients with stage I disease and high pre-DNA would also receive CCRT which was decided by clinicians. Neoadjuvant or adjuvant chemotherapy was cisplatin with 5-fluorouracil (PF), cisplatin with toxoids (TP), or cisplatin with both 5-fluorouracil and taxoids (TPF) (every three weeks; two or three cycles). CCRT was weekly cisplatin (30–40 mg/m^2^) or 3-weekly cisplatin (80–100 mg/m^2^) on weeks 1, 4 and 7 of radiotherapy.

## Follow-Up and Statistical Analysi*s*

Patient follow-up was measured from first day of therapy to last examination or death. Patients were examined at least every three months during first two years, with follow-up examinations every six months thereafter until death. End-points (time to first defining event) were disease-free survival (DFS), overall survival (OS), loco-regional relapse-free survival (LRRFS) and distant metastasis-free survival (DMFS). DFS was the primary end-point in our study.

Receiver operating characteristic (ROC) curves were used to calculate cut-off value for pre-DNA and CCD based on DFS. Serum lactate dehydrogenase (LDH) were classified as described previously^14^,^15^. The Chi-square test was used to compare clinical characteristics. Life-table estimation was performed using the Kaplan-Meier method and log-rank test. The multivariate Cox proportional hazards model was used to estimate hazard ratios (HR) and 95% confidence intervals (CI). All statistical tests were two-sided; *P* < 0.05 was considered significant. STATA statistical package (STATA 12; StataCorp LP, College Station, Texas, USA) was used for all analyses.

## Results

### Cutoff Values for Pre-DNA, LDH and Cumulative Cisplatin Dose

In total, pre-DNA data was not available for 49/549 (8.9%) patients. Median pre-DNA was 700 copies/ml (interquartile range, 0–7650 copies/ml) for the remaining 500 (91.1%) patients. Based on ROC analysis, the cut-off value of pre-DNA and CCD for DFS was 1460 copies/ml (AUC, 0.691; sensitivity, 0.717; specificity, 0.635) and 240 mg/m^2^ (AUC, 0.506; sensitivity, 0.526; specificity, 0.538), respectively. The LDH cut-off value was 245 U/L (normal range, 109–245 U/L in our hospital), as reported previously^15^,^16^.

### Patient Baseline Characteristics

For the entire cohort, the male (*n* = 406)-to-female (*n* = 143) ratio was 2.8:1; median age was 44 years-old (range, 18–77 years-old). In total, 239/549 (43.5%) patients received weekly cisplatin and 310/549 (56.5%) received 3-weekly cisplatin. The median cumulative cisplatin dose for all 549 patients was 160 mg/m^2^ (range, 25–300 mg/m^2^). In total, 457/549 (83.2%) patients received a CCD < 240 mg/m^2^ (group A) and 92 (16.8%), ≥240 mg/m^2^ (group B). The group B had a higher percentage of stage T4 (*P* = 0.037) and IV (*P* = 0.018) disease compared with that of group A. Moreover, more patients received 3-weekly cisplatin in group B than group A (*P* = 0.005; Table 1).

### Patterns of Treatment Failure

Median follow-up time for the cohort was 49.8 months (range, 1.3–68.4 months). Up to the last day of follow-up, 26/457 (5.7%) patients in group A and 4/92 (4.3%) in group B developed local failure (*P* = 0.606). 18/457 (3.9%) patients in group A and 0/92 (0%) patients in group B experienced regional failure (P = 0.01), and 53/457 (11.6%) in group A and 8/92 (8.7%) in group B experienced distant metastasis (*P* = 0.419). Moreover, 49/457 (10.7%) patients in group A and 7/92 (7.6%) in group B died (*P* = 0.368, Table 2). The majority of deaths were attributed to NPC.

### Prognostic Value of Cumulative Cisplatin Dose

**Figure 1:**
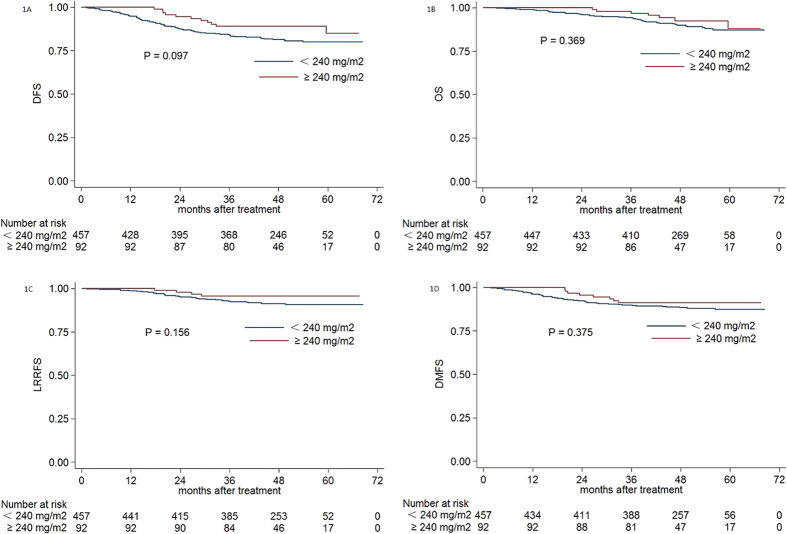
Kaplan-Meier DFS **(A)**, OS **(B)**, LRRFS **(C)** and DMFS **(D)** curves for patients with NPC stratified as the cumulative cisplatin dose <240 mg/m^2^ and ≥240 mg/m^2^ group. Abbreviations: DFS = disease-free survival; OS = overall survival; LRRFS = local-regional relapse-free survival; DMFS = distant metastasis-free survival.

The estimated four-year DFS, OS, LRRFS and DMFS rates for the entire cohort were 82.6%, 90.4%, 91.9% and 88.9%, respectively. For patients with a CCD ≥ 240 mg/m^2^ vs. < 240 mg/m^2^, estimated 4-year DFS, OS, LRRFS, and DMFS were 89.1% vs. 81.3% (*P* = 0.097, Fig. 1A), 92.4% vs. 90.0% (*P* = 0.369, Fig. 1B), 95.6% vs. 91.2% (*P* = 0.156, Fig. 1C), and 91.3% vs. 88.4% (*P* = 0.375, Fig. 1D), respectively.

Multivariate analysis was performed to adjust for potential prognostic factors, including gender, age, pathological type, smoking, drinking, pre-DNA, serum LDH, cisplatin regimen (weekly vs. 3-weekly), T category and N category. CCD was an independent prognostic factor for DFS (HR, 0.515; 95% CI, 0.267–0.995; *P* = 0.048; Table 3) in the era of IMRT.

### Subgroup Analysis Based on Pre-DNA

**Figure 2:**
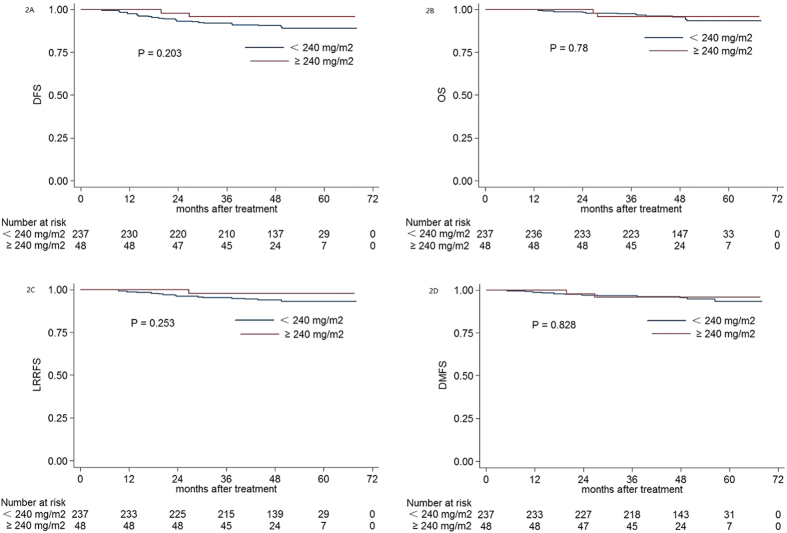
Kaplan-Meier DFS **(A)**, OS **(B)**, LRRFS **(C)** and DMFS **(D)** curves for NPC patients with pre-DNA < 1460 copies/ml stratified as the cumulative cisplatin dose <240 mg/m^2^ and ≥240 mg/m^2^ group. Abbreviations: Pre-DNA = pre-treatment EBV DNA; DFS = disease-free survival; OS = overall survival; LRRFS = local-regional relapse-free survival; DMFS = distant metastasis-free survival.

Among the 285/500 (57%) patients with pre-DNA < 1460 copies/ml, the median CCD was 160 mg/m^2^ (range, 25–300 mg/m^2^). Estimated 4-year DFS, OS, LRRFS, and DMFS rates for patients with a CCD ≥ 240 mg/m^2^ vs. < 240 mg/m^2^ were 95.8% vs. 90.6% (*P* = 0.203, Fig. 2A), 95.8% vs. 95.6% (*P* = 0.78, Fig. 2B), 97.9% vs. 93.9% (*P* = 0.253, Fig. 2C), and 95.8% vs. 95.5% (*P* = 0.828, Fig. 2D), respectively. Multivariate analysis indicated CCD was not an independent prognostic factor for DFS (HR, 0.405; 95% CI, 0.096–1.712; *P* = 0.219), OS (HR, 0.867; 95% CI, 0.194–3.878; *P* = 0.852), LRRFS (HR, 0.326; 95% CI, 0.043–2.467; *P* = 0.278) or DMFS (HR, 0.927; 95% CI, 0.205–4.190; *P* = 0.921) for patients with pre-DNA < 1460 copies/ml.

**Figure 3:**
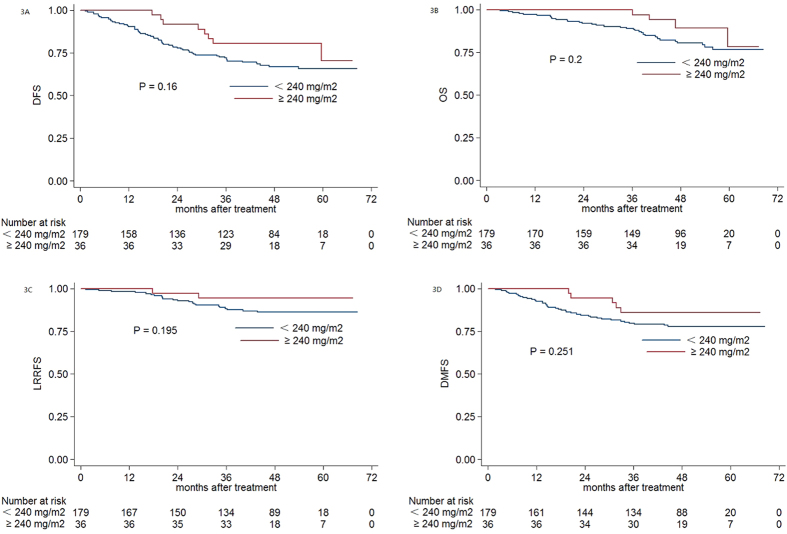
Kaplan-Meier DFS **(A)**, OS **(B)**, LRRFS **(C)** and DMFS **(D)** curves for NPC patients with pre-DNA ≥ 1460 copies/ml stratified as the cumulative cisplatin dose <240 mg/m^2^ and ≥240 mg/m^2^ group. Abbreviations: Pre-DNA = pre-treatment EBV DNA; DFS = disease-free survival; OS = overall survival; LRRFS = local-regional relapse-free survival; DMFS = distant metastasis-free survival.

For the 215/500 (43%) patients with pre-DNA ≥ 1460 copies/ml, the median CCD was 160 mg/m^2^ (range, 40–300 mg/m^2^). Estimated 4-year DFS, OS, LRRFS, and DMFS for patients with a CCD ≥ 240 mg/m^2^ vs. < 240 mg/m^2^ were 80.6% vs. 67.0% (*P* = 0.16, Fig. 3A), 89.4% vs. 80.6% (*P* = 0.2, Fig. 3B), 94.4% vs. 86.2% (*P* = 0.195, Fig. 3C), and 86.1% vs. 77.8% (*P* = 0.251, Fig. 3D), respectively. In multivariate analysis, CCD had no prognostic value for DFS (HR, 0.549; 95% CI, 0.261–1.154; *P* = 0.114), OS (HR, 0.454; 95% CI, 0.160–1.287; *P* = 0.138), LRRFS (HR, 0.397; 95% CI, 0.093–1.688; *P* = 0.211) or DMFS (HR, 0.566; 95% CI, 0.223–1.437; *P* = 0.231) for patients with pre-DNA ≥ 1460 copies/ml.

## Discussion

This current study demonstrated a CCD ≥ 240 mg/m^2^ is associated with significantly improved DFS among patients with NPC receiving only cisplatin during CCRT, which was consistent with the results of previous studies^9^,^11^. Moreover, no definitely prognostic association was found between CCD and pre-DNA.

Since group B had a higher percentage of stage T4 and IV disease, patients in that group therefore received a higher CCD compared with that of patients in group A. Moreover, the results of this current study revealed that a higher CCD had no benefit in terms of DMFS. The reasonable explanation is that cisplatin delivered during CCRT increases the effect of radiotherapy but does not reduce the risk of distant metastasis, which has been recommended by the NCCN guidelines. Therefore, the DFS benefit of a higher CCD may be attributed to increased regional control. Due to the relatively insufficient follow-up time, no significant difference in OS could be detected when the patients were stratified by CCD.

In the prospective NPC-9901 and NPC-9902 trials^17^, at least two cycles of cisplatin (100 mg/m^2^) improved local-free survival (LFS) and OS compared with one cycle. Moreover, 200 mg/m^2^ cisplatin was reported as an appropriate cumulative dose in other retrospective studies of NPC^9^,^10^ and head and neck cancer (HNSCC)^8^,^18^. It seemed that 200 mg/m^2^ has been used as the standard lowest cumulative dose in NPC and HNSCC. However, more recently, Ou *et al.* reported a total cisplatin dose > 300 mg/m^2^ was an independent prognostic factor for OS, DFS and DMFS in local advanced NPC^11^. Notably, a proportion of patients in this study also received induction or adjuvant chemotherapy. However, several prospective clinical trials have proven patients with locoregionally advanced NPC do not benefit from induction^19^,^20^,^21^,^22^,^23^ or adjuvant chemotherapy^24^. Therefore, the lowest effectively CCD in this study may have been inflated by delivering cisplatin-based induction or adjuvant chemotherapy^11^.

Notably, the CCD cutoff value of 240 mg/m^2^ did not have prognostic value in subgroup analysis based on pre-DNA, indicating that this cutoff value may be not appropriate for patients with pre-DNA higher or lower than 1460 copies/ml. The effective CCD may be higher in patients with pre-DNA < 1460 copies/ml and lower for patients with pre-DNA > 1460 copies/ml, or no definite relationship may exist between CCD and pre-DNA. Since no related studies have investigated this relationship, we therefore could not make a conclusion about the relationship between CCD and pre-DNA only based on the negative results of this current study. Future prospective studies are warranted to investigate the relationship further.

The optimal cisplatin schedule for CCRT in NPC remains under debate: is the weekly or 3-weekly regimen more effective? A third planned dose of 100 mg/m^2^ cisplatin was omitted in a substantial number of patients with HNSCC^8^. Moreover, in the NPC-9901 trial^5^, only 52% of patients received ≥three cycles of 100 mg/m^2^ cisplatin. This poor compliance may constrain the wide-spread use of 3-weekly cisplatin (100 mg/m^2^). The weekly cisplatin (40 mg/m^2^) regimen was first reported in 2002 by Chan *et al.*^25^. Loong *et al.* observed a significant association between >five concurrent weekly cycles of 40 mg/m^2^ cisplatin and better OS in subgroup analysis of 142 patients with stage II-III NPC^10^, and other retrospective studies also support the weekly strategy^26^,^27^,^28^. This current study also observed no significant prognostic differences between of patients receiving the weekly or 3-weekly cisplatin regimens, which further prove the efficacy of weekly regimen.

The purpose of concurrent chemotherapy is to achieve prognosis improvement with minimal and acceptable toxicities. Therefore, it is of great importance to establish the optimal dose of cisplatin administer during RT to gain survival benefit and avoid great toxicities. We identified a cumulative cisplatin dose ≥240 mg/m^2^ was an independent prognostic factor for DFS in patients with NPC receiving only cisplatin during CCRT. Therefore, in the clinic, at least six cycles of 40 mg/m^2^ may be considered when delivering the weekly cisplatin regimen, whereas three cycles of 80 mg/m^2^ may be adequate for the 3-weekly regimen.

This study is limited by its retrospective nature and a relatively short follow-up time, though we selected DFS as the major end-point to address these limitations. Moreover, the sample bias may existed because the data was from a single institution. However, it should not influence the results because we recruited all the eligible patients and the cohort was big. Further prospective studies of larger cohorts are warranted to confirm these results and further define the relationship between pre-DNA and the CCD to deliver more individualized therapeutic regimens in NPC.

## Conclusions

In this current study, a cumulative cisplatin dose ≥240 mg/m^2^ was identified as an independent prognostic factor for DFS in patients with NPC receiving single-agent cisplatin-based CCRT. Therefore, either weekly 40 mg/m^2^ cisplatin or 3-weekly 80 mg/m^2^ cisplatin could be appropriate standard regimens for patients with NPC receiving CCRT based on IMRT, during which a cumulative cisplatin dose of ≥240 mg/m^2^ should be delivered.

## Additional Information

**How to cite this article**: Peng, H. *et al.* The Cumulative Cisplatin Dose Affects the Long-Term Survival Outcomes of Patients with Nasopharyngeal Carcinoma Receiving Concurrent Chemoradiotherapy. *Sci. Rep.* **6**, 24332; doi: 10.1038/srep24332 (2016).

## Figures and Tables

**Table 1:** Baseline Characteristics of the 549 Patients Receiving Concurrent Chemoradiotherapy.

Characteristics	Cumulative	dose	Total	P^a^
	<240 mg/m^2^	≥240 mg/m^2^		
	No. (%)	No. (%)		
Gender				0.802
Male	337 (73.7)	69 (75)	406	
Female	120 (26.3)	23 (25)	143	
Age (years)				0.144
<50	318 (69.6)	71 (77.2)	389	
≥50	139 (30.4)	21 (22.8)	160	
WHO pathology				0.307
Type I	1 (0.2)	1 (1.1)	2	
Type II/III	456 (99.8)	91 (98.9)	547	
Smoking				0.283
Yes	157 (34.4)	37 (40.2)	194	
No	300 (65.6)	55 (59.8)	355	
Drinking				0.082
Yes	54 (11.8)	17 (18.5)	71	
No	403 (88.2)	75 (81.5)	478	
EBV DNA (copies/ml)^b^				0.977
<1460	237 (57.0)	48 (57.1)	285	
≥1460	179 (43.0)	36 (42.9)	215	
LDH (U/L)				0.631
<245	443 (96.9)	90 (97.8)	533	
≥245	14 (3.1)	2 (2.2)	16	
Cisplatin regimen				0.005
Weekly	212 (46.4)	28 (30.4)	240	
3-week	245 (53.6)	64 (69.6)	309	
T classification^c^				0.037
T1	79 (17.3)	21 (22.8)	100	
T2	74 (16.2)	12 (13.0)	86	
T3	255 (55.8)	41 (44.6)	296	
T4	49 (10.7)	18 (19.6)	67	
N classification^c^				0.583
N0	74 (16.2)	15 (16.3)	89	
N1	292 (63.9)	59 (64.1)	351	
N2	70 (15.3)	11 (12.0)	81	
N3	21 (4.6)	7 (7.6)	28	
Overall stage^c^				0.018
I	11 (2.4)	4 (4.3)	15	
II	112 (24.5)	27 (29.4)	139	
III	266 (58.2)	38 (41.3)	304	
IV	68 (14.9)	23 (25.0)	91	

Abbreviations: WHO = World Health Organization; EBV = Epstein-Barr Virus; LDH = lactate dehydrogenase.

^a^*P-*values were calculated using the Chi-square test or Fisher’s exact test if indicated.

^b^49 patients had no pre-treatment EBV DNA data.

^c^According to the 7^th^ edition of the AJCC/UICC staging system.

**Table 2:** Patterns of Treatment Failure for NPC patients with cumulative cisplatin dose <240 mg/m^2^ vs. ≥ 240 mg/m^2^.

Failure patterns	Cumulative	dose	P^a^
	<240 mg/m^2^	≥240 mg/m^2^	
	No. (%)	No. (%)	
Local only	15 (3.3)	2 (2.2)	0.496
Local + regional	3 (0.7)	0 (0)	1.000
Local + distant	5 (1.1)	2 (2.2)	0.46
Local + regional + distant	3 (0.7)	0 (0)	1.000
Regional only	9 (2.0)	0 (0)	0.061
Regional + distant	3 (0.7)	0 (0)	1.000
Distant only	42 (9.2)	6 (6.5)	0.35
Total distant	53 (11.6)	8 (8.7)	0.419
Total locoregional	39 (8.5)	4 (4.3)	0.173
Total failure	81 (17.7)	10 (10.9)	0.115
Total death	49 (10.7)	7 (7.6)	0.368

Abbreviations: NPC = nasopharyngeal carcinoma.

^a^*P* values were calculated using Chi-square test or Fisher exact test if indicated.

**Table 3:** Multivariate Analysis of Prognostic Factors Associated with Clinical Outcomes.

Endpoint	Variable	P^a^	HR	95% CI for HR
DFS	N classification	0.016	1.717	1.105–2.667
	Cumulative dose	0.048	0.515	0.267–0.995
	EBV DNA	<0.001	3.659	2.315–5.783
OS	N classification	0.033	1.851	1.051–3.260
	Age	0.026	1.843	1.076–3.158
	EBV DNA	<0.001	3.298	1.800–6.041
LRRFS	EBV DNA	0.013	2.228	1.183–4.196
DMFS	EBV DNA	<0.001	4.725	2.585–8.638

Abbreviations: DFS = disease-free survival; OS = overall survival; LRRFS = locoregional relapse-free survival; DMFS = distant metastases-free survival; HR = hazard ratio; CI = confidence interval.

^a^Multivariate *P*-values were calculated using an adjusted Cox proportional-hazards model. The following parameters were included in the Cox proportional hazards model with backward elimination: gender (male vs. female), age (≥50 y vs. <50 y), pathological type (type I vs. type II/III), smoking (yes vs. no), drinking (yes vs. no), EBV DNA (≥1460 copies/ml vs. <1460 copies/ml), LDH (≥245 U/L vs. <245 U/L), cisplatin regimen (weekly vs. 3-week), T category (T_1–2_ vs. T_3–4_), N category (N_0–1_ vs. N_2–3_), cumulative cisplatin dose (≥240 mg/m^2^ vs. <240 mg/m^2^).
